# Impact of a Regional Campus on the Placements of Students at Rural Pharmacy Experiential Sites

**DOI:** 10.3390/pharmacy9040195

**Published:** 2021-12-07

**Authors:** Stephanie Kiser, Elizabeth Ramsaur, Charlene R. Williams

**Affiliations:** 1 The Eshelman School of Pharmacy, The University of North Carolina Chapel Hill, Asheville, NC 27599, USA; stephanie_kiser@unc.edu; 2 Mission Health System, Asheville, NC 28801, USA; elizabeth.ramsaur@HCAHealthcare.com

**Keywords:** rural, experiential education, regional pharmacy campus

## Abstract

Pharmacist shortages in rural communities underscore the need to focus on increasing the pipeline of pharmacists practicing rurally. Experiential placement in rural communities is one method to approach this challenge. Regional pharmacy campuses may facilitate rural experiential placements. The objective of this study was to assess the effect of a regional campus on the number of rural experiential placements. This retrospective analysis compared experiential student placements in the five-year periods before and after the addition of a regional school of pharmacy campus. Experiential placements in the designated time periods were compared with respect to numbers of overall pharmacy practice experiences, experiences in rural locations, and rural counties with rotation sites. The average distance to rural sites was also compared. Differences in rural experiential placements were not statistically different. The number of rural counties with pharmacy experiential placements grew from eight to twelve, and driving distance increased. While institution of a regional campus contributed to an increase in the number of rural counties with experiential placements, overall rural experiential placements did not statistically differ versus suburban placements. Additional inquiry into factors that affect rural placement is needed to influence strategies to develop and maintain rural experiential sites and consistently place students at those sites.

## 1. Introduction

Rural health care provider shortage is a global concern [1,2]. The profession of pharmacy is no exception. The shortage of pharmacists in rural areas is compounded as rural pharmacies and hospitals close. Data from the Rural Policy Research Institute demonstrated a 16.1% decline in the number of rural pharmacies in the United States of America (USA) from 2003 to 2018, and 137 rural hospitals in the USA have closed from 2010 to 2021 [3,4]. Closure of smaller acute care sites or the loss of services in rural locations has also been noted as a concern in Australia, Canada, Germany, and France [5]. Policy makers, health professions schools, and public health agencies have striven to determine factors that may increase the supply and retention of rural healthcare workers [1,6,7]. One of the strategies suggested is to train students in rural practice sites [1]. 

Outcome data on the impact of rural placements on the supply and retention of pharmacists are relatively lacking. A survey of student pharmacists in New Zealand after exposure to a rural pharmacy practice experience found that the intervention increased the number of students who would consider rural practice [8]. Furthermore, rural placements of health professions students in Tasmania, Australia for a length of five days showed a trend to influence the intention to practice rurally; however, results were not statistically significant [9]. While more data is needed in pharmacy, some evidence in other health professions suggests that completing experiential training at rural sites influences future practice in those areas, as well as improves the ability of those trainees to manage issues specific to rural health [1,7,10,11]. 

Preceptors in rural regions are needed to implement rural placements. A national survey of volunteer pharmacy preceptors in the United States, published in 2008, revealed that 90% of the respondents were from urban or large population areas [6]. The authors implored college/school leaders to recruit more preceptors from rural areas in order to meet the demand for experiential sites and expose students to pharmacy practice in rural areas which are known to have pharmacist shortages [6]. These views align with the thoughts of rural pharmacists in Australia who participated in a qualitative survey; they identified the need for rural/regional pharmacy schools to support a pipeline of pharmacists in rural areas, as they believed that students were more likely to return to rural communities if they had trained in a regional pharmacy school [12]. Improving the recruitment and retention of pharmacists in specific regions of a state, such as rural areas, is one of the reasons cited for establishing a multi-campus program according to colleges and schools of pharmacies in the USA with these programs [13]. However, it is unknown if establishing a regional campus affects the number of rural experiential placements.

The primary purpose of this study was to evaluate the number of experiential placements in the Western North Carolina (WNC) region of North Carolina, before and after the establishment of a regional campus in that area. 

## 2. Materials and Methods

**Figure 1:**
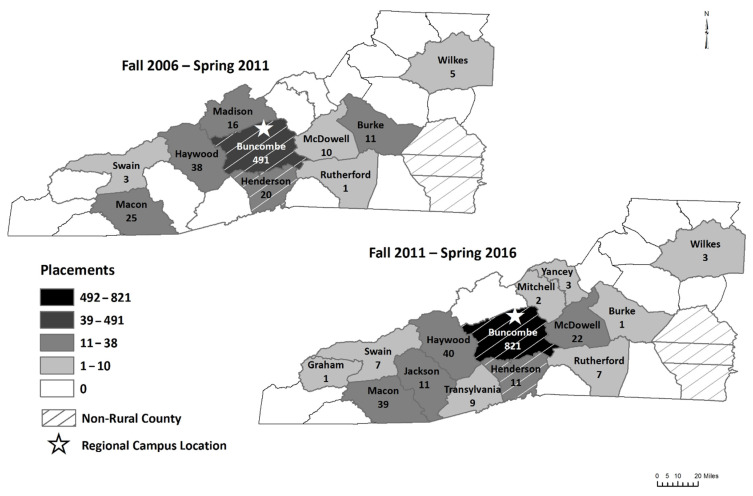
Student Pharmacist Experimential Placements in Western North Carolina by Country 2006–2011 Compared to 2011–2016. Map created using ArcGIS® software by Environmental Systems Research Institute (Esri) by Joan Colburn, MLIS, UNC Health Sciences at MAHEC, 12 June 2021.

This quasi-experimental study was a retrospective, quantitative analysis of experiential placement data spanning the five-year periods immediately before and after the University of North Carolina (UNC) Eshelman School of Pharmacy’s Asheville regional campus opened. This study was deemed as nonhuman subjects research by the University’s Institutional Review Board (IRB) and thus did not require approval. De-identified student placements in introductory and advanced pharmacy practice experience (IPPE, APPE) sites in WNC obtained from the school’s experiential management database were compiled and analyzed. In this study, WNC was defined as the 23 most western located counties in North Carolina geographically (Figure 1). The data included student experiential placements from Fall 2006 to Spring 2011 (before the campus opened) and from Fall 2011 to Spring 2016 (after the campus opened) (Figure 1). It should be noted that the majority of student placements in the WNC region are from the Asheville campus, though some students from the Chapel Hill campus are also placed in WNC. 

Source data included pharmacy practice experience date, experience location, and rotation type (community IPPE, health system IPPE, community APPE, health system APPE, inpatient APPE (excluding health system APPE and nonpatient care elective APPE), ambulatory care APPE, non-patient care elective APPE). At the time of the study, students completed two, month-long IPPEs in a hospital (health system IPPE) or a community pharmacy setting (community IPPE). One IPPE was completed at the end of the first professional year, and one was performed at the end of the second professional year. During the fourth and final professional year, students completed nine, one-month APPEs. At the time of the study, students were required to have one APPE in a community pharmacy environment (community APPE), one in a hospital setting focused on advanced distribution and operations (health system APPE), one in an outpatient primary care setting (ambulatory care APPE), one in a generalized acute care setting such as general medicine, internal medicine, or family medicine (classified as inpatient APPE in the study), and one in a specialized acute care setting such as cardiology, critical care, emergency medicine, or others (also classified as inpatient APPE for study purposes given the limited availability of these rotations in rural areas at the time of the study). The remaining four rotations were electives. Students could request up to two non-direct patient care APPE electives such as industry, managed care, academia, or other non-direct patient care APPEs. Otherwise, the elective APPEs were scheduled in the aforementioned direct patient care areas. Students could submit preferences for rotations, but selection based on preferences was not guaranteed. The county where the rotation took place was identified for each site, and then further designated as either suburban or rural according to the county’s designation by the North Carolina Rural Center (of note, there are no urban counties in Western North Carolina) [14]. 

Descriptive statistics were used to analyze the data using Microsoft^®^ Excel (version 15). The time periods were compared with respect to the number of overall placements and the number and percentage of placements in rural locations. A Chi-square test, with Fisher’s exact test, where appropriate (Social Science Statistics, 2021), was used to assess differences in the number of student experiential placements in rural and suburban areas overall, per county, and by rotation type before and after the arrival of the regional campus. The a priori level of significance was less than 0.05. The rurality of the represented counties remained consistent during the study period.

Finally, the distance from the regional campus to each rural site was determined using an online navigation tool (Google Maps, 2020) so that the average distance in the before and after time periods could be compared. The driving distance was reported as mean (standard deviation (SD)). An independent t-test was used to compare differences in the driving distance before and after the establishment of the regional campus (GraphPad Prism, 2021). The a priori level of significance was less than 0.05. Students were expected to commute to sites within 60 miles (96.6 km) and were not placed at sites beyond 60 miles unless housing was provided. 

## 3. Results

The results show that the number of student placements in pharmacy practice experiences sites in WNC overall increased from 620 (in the five-year period before the regional campus opened) to 977 (in the five-year period after) (Table 1). The difference in overall rural placements before and after the campus was not statistically significant (Table 1). There were no statistically significant differences in placements in rural versus suburban areas per rotation type before and after the regional campus was established except for communityAPPEs, which were significantly decreased from 21 to 14 in rural areas (x^2^ (1, N = 1632) = 6.44, *p* = 0.011).

During the five years prior to the opening of the regional campus, there were student placements in eight rural WNC counties, compared to 12 rural counties in the five years after the regional campus opened. Students were placed in six rural counties (Graham, Jackson, Mitchell, Rutherford, Transylvania, Yancey) for the first time after the regional campus opened. Madison County had student placements in the time before, but not after, the campus opened (Figure 1). On the individual county level, there were four rural counties with statistically significant differences (Fisher’s exact test) in the number of student placements before and after the campus opening. Student placements decreased in Madison and Burke counties (*p* < 0.05) and increased in Rutherford and Jackson counties (*p* < 0.05). One of two suburban counties, Henderson, had a statistically significant decrease in placements between the two time periods (*p* < 0.05).

Before the regional campus opened, students placed in rural rotation sites had to travel an average of 45.2 (26.3) miles (72.7 (42.3)) kilometers) get to their rotation sites. After the regional campus opened, this distance increased to a mean of 54.1 (22.2) miles (87.1 (35) kilometers) (*p* = 0.0031).

## 4. Discussion

This study describes the impact of the establishment of a regional school of pharmacy campus in North Carolina, USA on experiential placements. Due to the paucity of literature for pharmacy related to this topic, one of the strengths of this study is the contribution of data in this space that may be useful to other schools with regional campuses and rural experiential sites. As colleges and schools of pharmacy educate and train the future workforce, it is imperative to examine the opportunities and challenges associated with increasing experiential placements in rural settings. Predicted shortages of primary care physicians and closures of critical access hospitals in rural communities are expected to worsen already existing health disparities [15,16]. Experiential education in schools and colleges of pharmacy primarily takes place in urban areas or academic medical centers [6] and, unlike experiential education in medical schools, rarely emphasizes the provision of patient care in rural U.S. communities, where chronic diseases are prevalent and many residents struggle with poverty and poor access to healthcare. As colleges and schools of pharmacy address health workforce shortages and health disparities in rural communities, experiential placement can be a positive and viable method to use for increasing health science students’ intentionality to take up rural practice [9]. In addition to rural experiential placement, establishing a regional pharmacy campus in a mostly rural part of the state is another strategy to employ to further facilitate exposure of learners to rural practice while also addressing the health needs of these communities.

This study provides valuable guidance that despite increasing the number of rural counties with experiential placements after the establishment of the regional campus, there was not a statistical difference in the percentage of rural experiential placements. One possible reason for this finding is the concurrent growth of suburban placements. Strengthened partnerships with sites in suburban areas facilitating placement of students at those sites after the establishment of the regional campus may have also contributed to the results. While experiential placements in some rural counties increased, they decreased in others. This could have been due to changes in preceptor availability, rotation type availability, student preferences, or other factors not captured in this study. Further research is needed to understand differences in county experiential placements. Using a design thinking framework to understand barriers of student placement in rural locations, Wolcott and colleagues found that students, practitioners, and administrators identified perceived disadvantages of rural experiences such as isolation, lack of housing, greater commuting distance, and lack of specialties [17]. Examples of strategies that participants identified included ideas for connection (community champion for the region to support connection), well-being (workshop on culture intelligence), and housing and commuting solutions (recreation vehicles) [17]. There are additional approaches and deeper understanding needed relating to the barriers impeding and factors promoting the placement of students in rural experiential sites that should be explored in future studies. Further inquiry is also needed to determine the impact of rural experiential placement on post-graduate practice settings.

Considering how to improve upon the findings, it may be helpful to seek guidance from medical education colleagues who have a larger footprint of literature and success in this space. An article by Greenhill et al. describes how the establishment of rurally placed clinical schools and practice sites in Australia has positively influenced the supply and retention of rural primary care in remote areas [18]. They mention the creation of good infrastructure and support for rural providers as keys to success, which could easily be applied to other health professions globally [18].

UNC Eshelman School of Pharmacy has begun to develop key infrastructure through the establishment of the regional campus, placing the school much closer to rural preceptors and sites in WNC, while creating key support for students. Building upon that initial, important step, continued efforts are needed to foster relationships, connections, and support for rural experiential sites, while also identifying new sites across the region. Providing accessible professional development opportunities for preceptors in rural regions is a key component of that support. Since the completion of this study, the school has been focusing on providing more virtual resources to increase accessibility. Future efforts will be aimed at offering additional opportunities for development and relationship building during site visits. Electronic resources and bringing development programming to sites are general strategies not necessarily targeted to solely rural preceptors that other experiential programs have used as a means to increase engagement and strengthen relationships with sites and preceptors [19].

In addition to supporting preceptors, it is important to understand the key incentives that might draw pharmacy students to explore rural experiential sites and remove any identified barriers. The study results revealed that since the regional campus was established, students are traveling slightly further to their experiential sites. In efforts to grow the regional rural experiential footprint, students may have been inadvertently disadvantaged as it relates to travel distance. Future inquiry is needed to understand this barrier which can influence the development of infrastructure and support for students. The number of counties with rural sites may be growing, but utilization of those sites could be limited due to the lack of consistent housing options in more distant rural areas. Other areas of inquiry include determining factors that guide experiential coordinators’ student placement decisions in rural areas (e.g., student preferences, quality assurance, familiarity with sites and preceptors, preceptor availability, rotation type availability, scheduling logistics, housing availability, etc.). Collaboration with other regionally placed health sciences campuses has recently begun with a focus on increased student housing options and interprofessional education experiences in rural communities. These areas will be included in a future study of this issue.

This study has several limitations. First, this study was completed at one school of pharmacy in North Carolina, USA which may limit the generalizability of findings. Future studies in other parts of the USA and globally are needed. Second, factors affecting student placement each year were not recorded such as changes in preceptor and site availability, rotation type availability, student preferences for placements, housing availability and usage, efforts to meet accreditation quality assurance standards that may have affected utilization, and increased use of experiential faculty hired at partnering sites in suburban areas of the region to support the educational initiatives of the campus. Further inquiry is needed around the factors that affect experiential placements in rural areas including student perceptions. Lastly, changes in rural placements for the rest of the state were not evaluated.

## 5. Conclusions

This study indicates that the establishment of a regional pharmacy campus appears to have some positive influence on the number of counties with rural experiential placements in the area surrounding the campus. In contrast, the overall percentage of rural experiential placements did not change across the time period evaluated. Further study is needed to identify and evaluate barriers impacting student rural experiential placements.

## Figures and Tables

**Table 1:** Number and percentage of student placements in suburban and rural counties in Western North Carolina before and after the establishment of the regional campus.

Type of Placement	2006–2011 Pre-Establishment N (%)	2011–2016 Post-Establishment N (%)
Suburban	511 (82.4)	832 (85.2)
Rural	109 (17.6)	145 (14.8)
x^2^ (1, N = 1597) = 2.13, *p* = 0.144		

## Data Availability

Data available on request due to restrictions (education records).
